# Validation and Psychometric Evaluation of the COVID-19 Risk Perception Scale (CoRP): a New Brief Scale to Measure Individuals’ Risk Perception

**DOI:** 10.1007/s11469-021-00660-6

**Published:** 2021-09-27

**Authors:** Vincenza Capone, Anna Rosa Donizzetti, Miriam Sang-Ah Park

**Affiliations:** 1 Department of Humanities, University of Naples “Federico II”, Via Porta Di Massa, 1 80133 Naples, Italy; 2 School of Social & Health Sciences, Leeds Trinity University, Horsforth, Leeds UK

**Keywords:** Risk perception, Scale validation, COVID-19, Mental health, Health promotion, CoRP (Covid-19 Risk Perception Scale)

## Abstract

The aim of the work was to develop and validate the COVID-19 Risk Perception Scale (CoRP), a brief self-report questionnaire for individuals’ perceptions of risk in the COVID-19 pandemic. Two studies were conducted in order to evaluate the new scale’s psychometric properties. Study 1 included 269 Italian participants (77.3% female) to initially test the scale’s structure and construct validity. Study 2 involved 1061 (76.2% female) Italians aged 18 to 80 years old and examined the structure of the scale, construct validity, and age invariance. Exploratory and Confirmatory factor analyses confirmed the one-factor solution, and the structure of the scale was found to be invariant across age groups. The scale also demonstrated a high internal reliability. The CoRP correlated positively with the fear of COVID-19 scale, and low with the Impact of Event and distressing phenomena as measured by GHQ. The present work thus affirms that the CoRP is a valid instrument for measuring individuals’ risk perception of COVID-19.

## COVID-19

During the last few months of 2019 and the first quarter of 2020, a respiratory coronavirus disease (COVID-19) has suddenly become a major worldwide emergency. COVID-19 has affected individuals in 180 countries and on March 11, 2020, it was officially declared a global pandemic (WHO, 2020a). COVID-19 is a novel pathogen and has been characterized by high degrees of uncertainty, especially in its mode of infection and degree of fatality (Ruiu, 2020). With no vaccines or treatments developed specifically for the virus, avoiding the contact was considered the best existing way to prevent the infection in the initial months of the pandemic. Other further protective actions, such as frequent handwashing, avoiding touching nose, mouth, and eyes, and covering mouth and nose while coughing and sneezing, and wearing of mask were also encouraged (WHO, 2020b). A series of governmental decisions was introduced to restrict social and economic behavior, resulting in months-long lockdowns of educational and business activities in countries around the world. This has largely changed the life routine of individuals and the way the society in general functions.

Toward the end of the 2020, the vaccination program against COVID-19 started and substantially alleviated the problems related to the spread of the virus. Subsequently, the challenge for the policymakers is to encourage people to receive the vaccine and, at the same time, promote compliance with anti-contrast regulations. A recent study affirmed that as COVID-19 risk perception increased, so did the intention to receive the vaccine (Caserotti et al., 2021). Compliance with the prescribed behavioral norms and the implementation of preventive and protective measures are steered by the perception of risk related to the virus (Capone et al., 2020; Wise et al., 2020). As for flu vaccination, low risk-perception, doubts about the effectiveness of vaccines, and fear of side-effects were the most common reasons for rejection (Lehmann et al., 2014). Perceived risk of infection and precautionary behavior can vary through time, impacting the effectiveness of disease control measures (Caserotti et al., 2021). After all, risk perceptions refer to people’s intuitive evaluations of hazards that they are or might be exposed to, including a multitude of undesirable effects that people associate with a specific cause (Rohrmann & Renn, 2000).

## Risk perception and COVID-19

Risk perception is the subjective judgment that individuals create and hold regarding the characteristics, severity, and way in which a risk is managed and refers to individuals’ psychological evaluations of the probability and consequences of an adverse outcome (Sjöberg, 2000). It is a subjective psychological construct that is influenced by cognitive, emotional, social, cultural, and individual variation both between individuals and across countries (van der Linden, 2017). As Slovic (1992, p. 690) stated, “risk does not exist independent of our minds and culture”. In his works, Slovic (1992, 2000) demonstrated that number of people affected, dread, and knowledge were three important dimensions that could be used to describe risk perception. This approach has provided a solid foundation for describing how individuals orient toward a range of hazards. The epidemic has received broad media attention globally and is subjected to much discussion on social media, contributing to the types of information about the virus and influencing the perception of risk among people.

Risk perception is rightly placed as a core concept in theories explaining for beliefs and behaviors relating to health, such as Health Belief Model and Protection Motivation Theory (Zani & Cicognani, 2000). The literature has particularly emphasized on the role of risk perception in motivating health protection behaviors generally (Capone & Petrillo, 2010, 2012; Donizzetti, 2009; Floyd et al., 2000), and also especially during pandemics (Wise et al., 2020). It is known to be a significant determinant of the public’s willingness to cooperate and adopt safety behaviors (Dryhurst et al., 2020). Furthermore, public understanding of risk could be a determinant of community mental and physical health and well-being (Baldwin et al., 2020; Birley, 2015). This is the case especially during a pandemic, as compliance with recommended precautionary behaviors is not always evident. Considering factors influencing behavioral change during outbreaks of infectious diseases is, therefore, necessary. In particular, individuals’ assessments of the risk of the disease and the ways in which such assessments lead to the change in behavior is important (Sjöberg, 2000), as this could lead to reducing the spread of the disease.

Research has demonstrated that perceptions of risk for COVID-19 tend to vary across individuals and groups. Recent studies have highlighted that older people estimated the risk of COVID-19 to be less dangerous than younger people, and that women were more concerned about COVID-19 than men (Dryhurst et al., 2020; Gerhold, 2020). University students had the highest perceived threat level towards COVID-19 in comparison to other medical threats (Shabu et al., 2020). In China, social risk judgment was higher and life satisfaction was lower after the declaration of COVID-19 on January 20, 2020 (Li et al., 2020). Davico and colleagues (2020) affirmed that the psychological impact of COVID-19 resulted very strong in Italy and up to 30% of adults and children in the pandemic area were at a high risk for post-traumatic stress disturbances. In a recent study of COVID-19 risk perception (Shabu et al., 2020), participants who have had direct personal experience with the virus were found to perceive more risk compared to those who did not, and people who received information on the virus from family and friends perceived more risk.

As perceiving the risk of being infected has been identified as an important predictor of safety behaviors in the context of COVID-19 (Centers for Disease Control and Prevention 2020), there is a need for developing tools that can assess COVID-19 risk perceptions accurately and effectively. Different tools for measuring individuals’ risk perception of COVID-19 were recently developed in countries and regions including Vietnam (Huynh, 2020), China (Dai et al., 2020), Europe (Dryhurst et al., 2020; Gerhold, 2020), and North America (Dryhurst et al., 2020), with each of these studies having come up with ad hoc tools for their research aims. For example, Dai et al. (2020), in a study aimed to investigate the risk perception and immediate psychological state of health workers in the early stage of the COVID-19 epidemic, designed a tool ad hoc composed of 6 questions aimed to investigate the participants’ perceived seriousness of the COVID-19 (example item: “Are you worried about getting infected with COVID-19 yourself?”). Dryhurst et al.’ (2020) COVID-19 Risk Perception scale covers affective, cognitive, and temporal-spatial dimensions. The index, developed ad hoc for the study, included items capturing participants’ perceived seriousness of the COVID-19 pandemic, perceived likelihood of contracting the virus themselves, perceived likelihood of their family and friends catching the virus, and their present level of worry about the virus (example item: “How worried are you personally about the following issues at present?—Coronavirus/COVID-19”).

Although the importance of analyzing risk perceptions at pandemic times is thus recognized in many studies, to date, there is little work that has examined the psychometric characteristics of scales measuring perception to be at risk of COVID-19, and there is little agreement or consistency in the scales developed and used in the existing studies. A review of studies highlighted that the risk perception-behavior relationship was stronger for studies that had higher quality risk measures (Brewer et al., 2007), and a validated measure for COVID-19 risk perception that has strong psychometric properties is much needed.

## Aim and hypotheses

We aimed, therefore, to validate a brief tool, the COVID-19 Risk Perception Scale (CoRP, Italian Version), examining the structure, reliability, convergent, discriminant validity, and invariance across age groups. We hypothesized that the CoRP scale would have a high internal reliability and is one-dimensional. Rapid assessments are efficient methods for collecting information in a short period of time and when it is not possible to implement classical research methodologies as suggested in pandemic.

We hypothesized that CoRP should have good convergent validity, that it would correlate positively with a corresponding measure of Perceived Fear of COVID-19 (Study 1). Literature on past virus outbreaks has underlined the role of fear in exacerbating the harm perception of the infectious disease (Pappas et al., 2009). Referring to COVID-19, fear is inherent in its characteristics and is not completely manageable, especially with an excess of public concern around it (Ahorsu et al., 2020; Cori et al., 2020). The uncertainty and situational control would be strictly connected with the perception of its risk to the self and to the public (Lerner & Keltner, 2001).

Discriminant validity was assessed by examining the correlation of CoRP scale with measures of negative psychological reactions. We included Perceived Impact of Event as a measure of stress reactions after traumatic events (Study 1) and General Health Questionnaire (GHQ) as a measure of mental distress (Study 2). The concept of impact of events is related to risk perceptions, but the notions are different (Hershey et al., 1994). Understanding the impact of traumatic experiences on the thoughts and behaviors of people is different from their perception to be at risk. While risk perception is the subjective assessment of the probability of the occurrence of a specific type of adversity (Sjöberg, Moen, & Rundmo 2004), the impact of event refers to the degree of distress a person feels in response to traumatic event. We thus expected that these variables are very different from each other, highlighting low correlations. Likewise, there is no a priori reason to expect that individuals’ risk perception would be strongly correlated with general dysfunction. With regards to group invariance, we hypothesized that the functioning of the CoRP items would not differ across the age range.

## Construction of the CoRP

The instrument was developed in line with the methodology of psycho-social research on construction of measurement scales (DeVellis, 2003). We also analyzed the items of the developed scales on risk perception from COVID-19 in the aforementioned studies. Several theories have remarked on the importance of emotion dimension of risk perception, above all, in the first stage of a pandemic (Loewenstein et al., 2001; Schwarz & Clore 1983). However, the vast majority of literature on risk perception has recommended the inclusion of cognitive dimension which directly or indirectly characterize and influence people’s risk perception (Flesia et al., 2020; Slovic, 1987, 2001). This is because cognitive risk perception represents analytical information processing that is slow, cautious and sequential, and requires the use of more cognitive resource. Starting with these considerations and from an analysis of literature on risk perception, we decided to consider the *cognitive* dimension of risk perception, aimed to capture participants’ perceived seriousness of the COVID-19 pandemic, perceived likelihood of contracting the virus themselves and perceived likelihood of their family catching the virus. Slovic & Peters (2006) labeled cognitive dimension of the risk perception as the analytic system in which judgments arrived at through the application of logical connections, systematic comparison of evidence and information, and a conscious justification for action. Measurement approaches for this emphasized individuals’ identification and assessment of objective, observable properties of a hazard. The cognitive dimension of risk characteristics is associated with the probability and severity of consequences that are assessed from available information (Bonnet et al., 2012).

Relevant and possible items were pooled together by two researchers who were expert in health psychology. After removing items with overlapping content or expressions, 12 items were retained for further evaluation. An expert panel of health and social psychologists then evaluated the 12 items, and 4 items were deleted based on the suggestion from the panel. The retained 8 items were then sent out to a different expert panel (comprising a health education specialist, a social psychologist, and a sociologist) for a review. Four items were further omitted based on the comments from the expert panel. The final 4 selected items investigated individuals’ perception of risk to COVID-19 in this study (see Table 1).

**Table 1:** The selected items of the CoRP scale

Italian version	English version
CoRP_01 — *Quanto sei preoccupato di contrarre l’infezione da COVID-19?*	CoRP_01 — *Are you worried about getting diseased with COVID-19 yourself?*
CoRP_02 — *Sei preoccupato che i tuoi familiari possano contrarre l'infezione da COVID-19?*	CoRP_02 — *Are you worried about your family getting infected with COVID-19?*
CoRP_03 — *Sei preoccupato per l’inadeguatezza delle misure di protezione?*	CoRP_03 — *Are you worried about inadequate protective measure?*
CoRP_04 — *Sei preoccupato per l’attuale strategia di prevenzione e controllo del virus?*	CoRP_04 — *Are you worried about the current grassroots prevention and control strategy?*

Items were assessed on a five-point Likert scale (1 = *strongly worried*; 2 = *worried*; 3 = *not sure*; 4 = *not too worried*; 5 = *not worried at all*), with lower score indicating higher level of concern.

## Procedure and statistical analyses (Studies 1 and 2)

Research participation was subjected to privacy information and consent to the processing of personal data in accordance with the applicable regulations. The data were provided by individuals in Italy within the context of a broader study conducted within their organizations assessing efficacy. The data were collected first in April 2020 (Study 1) and May 2020 (Study 2). Ethical approval was obtained from the Department of Humanities Ethical Committee of Psychological Research prior to commencement of this project. All participants provided informed consent.

In both studies, the items of CoRP were evaluated with regards to variance and frequency distribution as a means to select the appropriate ones to be used in factor analysis. The Kaiser–Meyer–Olkin (KMO) measure of sampling adequacy and Bartlett’s test of sphericity were used to test whether the dataset was appropriate for factor analysis. The dimensionality of the scale was investigated using exploratory factor analysis. In order to facilitate the interpretation of the factor analysis, we followed the recommendations of Fabrigar et al. (1999) and performed a principal-axis factor analysis with promax rotation (Nunnally & Bernstein, 1995). For reliability, we used the analysis of internal consistency through covariance between items using Cronbach’s alpha. An internal consistency of greater than 0.70 is thought to be necessary for a valid psychological scale (Nunnally & Bernstein, 1994). In order to test for internal consistency, we calculated the corrected correlation between the score of the item and the total scale.

Confirmatory factor analysis (CFA) was then conducted using the maximum likelihood estimation method to evaluate the underlying structure of items. In order to evaluate the solution, we performed a tau-equivalent model (Steyer, 2001; Traub, 1994), which meant that the error variance for each item was to be different but all of them should equally explain for the true score variance. We took into account various goodness of fit indexes to evaluate the models: chi-square (*X*^2^), root mean square error of approximation (RMSEA), root mean square residual (RMSR), comparative fit index (CFI) and the Tucker–Lewis index (TLI). *X*^2^ tests the null hypothesis of perfect model fit where the residual covariance equals zero. CFI and TLI values above 0.90 (Bentler, 2005; Byrne, 1994), RMSEA values below or equal to 0.06, and SRMR values equal to or below 0.09 (Hu & Bentler, 1999) were considered adequate. Relations between the measures were examined using the Pearson product–moment correlations. Statistical significance was set at p-value < 0.05. Analyses were conducted with SPSS 21.0, and Lisrel 8.51 for CFA.

## Study 1: Analyses of the psychometric properties of the initial scale

### Method

#### Pre-test sample

In this study, the questionnaire was administered to 269 university students, (77.3% female), ranging in age between 18 and 45 years (mean age = 22.99 years old, SD = 2.78). All participants were Italian. 70% of participants declared that in the territory where they live, there were cases of people declared positive for COVID-19, and 9.5% said they knew them personally.

#### Measurements and procedures

A questionnaire was administrated to the participants with three scales and a form. Participants were recruited via e-mail campaigns, social media, and SMS campaigns. All participants involved voluntarily agreed to participate in the data collection procedure during the year 2020 (March–June). They were invited to fill out an online questionnaire connecting to a weblink associated to the Google Forms platform, an online application developed by Google for collecting data online. It was guaranteed to participants that their answers would be confidential and processed anonymously. There was no time limit for answering the questions; nevertheless, the questionnaire was completed in approximately 20 min. The scales were:

1) The CoRP scale;

2) The Fear of Covid (Graffigna et al., 2020) a single item measuring how much individual is afraid of Covid-19. The scale response ranged from 0 (*not at all*) to 10 (*extremely*);

3) The Impact of Event Scale-6 (IES-6; Italian Version, Giorgi et al., 2015) includes a total of six items, with two items from each of the three subscales, intrusion, hyperarousal and avoidance. Participants were asked to report their symptoms in the past 15 days on a Likert Scale ranging from 0 (*not at all*) to 4 (*extremely*). An example of the items is *“In the past 15 days, I felt watchful or on-guard”*. The internal reliability was α = 0.80;

4) Socio-demographic information: participants provided information relating to their gender, age and level of education.

### Results

In order to examine item quality and probability of dysfunctional items or polarization, we estimated the variances, means and standard deviations of the four CoRP items. Results shown in Table 2 indicate that all items have a normal distribution regarding the sample’s answers. The average score obtained in the CoRP was 2.42 (SD = 0.86). All item-test correlations were between 0.60 and 0.71, suggesting good psychometric properties. To further examine item quality, we carried out a correlational analysis between the four items. All of the inter-item correlations in the CoRP were positive and statistically significant (p < 0.001), ranging from r = 0.37 to 0.81.

**Table 2:** Mean, Standard deviations, Variance, Asymmetry and Kurtosis of the items (N = 267)

	Mean	Standard deviation	Variance	Asymmetry	Kurtosis
CoRP_01	2.94	1.130	1.278	0.094	− 1.042
CoRP_02	2.06	1.069	1.143	0.970	0.117
CoRP_03	2.27	1.020	1.040	0.682	− 0.076
CoRP_04	2.41	1.067	1.138	0.597	− 0.347

### Analysis of the dimensionality of the instrument

Bartlett’s sphericity test, which was equal to *X*^*2*^(*df* = *6, N* = 267) = 508.380, *p* < 0.00, and the Kaiser-Meyer–Olkin index (KMO) with a result of 0.657, guaranteed that the correlation matrix was suitable for exploratory factor analysis. One factor with eigenvalues greater than one emerged. It explained 53.54% of the variance (Table 3). We estimated the scale reliability using the Cronbach alpha index, which was 0.81.

**Table 3:** CoRP. Loading of the items, corrected correlations and reliability of the item (N = 267)

	Factor loading	Corrected correlation	α if deleted
CoRP_01	0.637	0.595	.79
CoRP_02	0.645	0.608	.78
CoRP_03	0.859	0.711	.73
CoRP_04	0.762	0.627	.77

### Convergent and discriminant validity

The CoRP converged positively with the Fear of Covid (*r* = 0.56, *p* = 0.000), demonstrating convergent validity. Regarding the discriminating validity, the CoRP correlated positively but very moderately with the Impact of Event (*r* = 0.37, *p* = 0.000).

## Study 2: Analysis of the psychometric properties of CoRP

### Method

#### Participants

A convenience sample of 1061 (76.2% female), ranging in age between 18 and 80 years (mean age = 37.30 years old, SD = 14.13) and living in Italy, participated in Study 2. 73% of participants declare that in the territory where they live there are cases of people declared positive for COVID-19, and 9.5% say they know them personally. 41.6% of participants were graduated. Many (41.2%) were married and working (55.2%), while others were university students (29.7%).

#### Measurements

A questionnaire was administrated to participants including the CoRP scale and the General Health Questionnaire-12 and sociodemographic variables. The General Health Questionnaire-12 (GHQ-12; Goldberg, 1992; Italian version by Piccinelli & Politi, 1993) is aimed at detecting common symptoms which are indicative of the various syndromes of mental disorder. The scale consists of 12 items rating with a 4-point rating scale, ranging from 1 (*strongly disagree*) to 4 (*agree*), and the internal reliability, for this study, was α = 0.88 in this study. An example item is *“Have you recently been able to concentrate on whatever you’re doing?”*.

### Results

Analysis of the CoRP individual items indicated that item scores were not skewed, with none of them showing extreme means and close to zero variances (Table 4). The average score obtained in the CoRP was 2.37, SD = 0.834.

**Table 4:** Properties of the items on the CoRP scale (N = 1061)

Item	Mean	Standard deviation	Variance	Asymmetry	Kurtosis
CoRP_01	2.79	1.109	1.231	0.274	− 0.789
CoRP_02	1.95	1.010	1.021	1.114	0.703
CoRP_03	2.36	1.090	1.187	0.547	− 0.542
CoRP_04	2.38	1.097	1.202	0.537	− 0.588

#### Analysis of the dimensionality of the instrument (internal structure)

In order to test for the internal consistency, we calculated the corrected correlation between the score of the item and the total CoRP. Coefficients were between 0.54 and 0.67 and were considered adequate since they were greater than 0.30. Once again, we followed the recommendations of Fabrigar et al. (1999) in the exploratory factor analysis and performed a principal-axis factor analysis, using promax oblique method. Bartlett’s sphericity test was equal to *X*^2^(*df* = 6, *N* = 1061) = 1434.562 (*p* < 0.001) and KMO index was 0.66, indicating that correlation matrix was adequate. Considering the results from the factor analysis, corrected correlations of the items with the entire scale and the reliability analysis, we did not drop any items. Maximum Likelihood (ML) estimation was used considering eigenvalues greater than 1, and we established that an adequate single-factor solution could be found (Table 5).

**Table 5:** CoRP. Loading of the items, corrected correlations and reliability of the item (N = 1061)

	Factor loading	Corrected correlation	α if deleted
CoRP_01	0.660	0.577	.73
CoRP_02	0.604	0.535	.75
CoRP_03	0.809	0.667	.68
CoRP_04	0.610	0.549	.74

Confirmatory factor analyses were conducted to ascertain the factor structure of the CoRP. Table 6 shows the single-factor structure: congeneric model, tau equivalent model, tau equivalent model with correlation between item 1 and 2 and item 3 and 4, and parallel model. The best fit indices were those of the equivalent tau model with the correlation of detection errors. The standardized regression coefficients weights of all variables loading onto the factor were between 0.50 and 0.88, with all critical ratios above 1.96 (which means that all the regressions were statistically significant at the 95% confidence level).

**Table 6:** Confirmatory factor models of theories of the latent structure of the CoPR items

Model	χ^2^ (df); p	RMSEA (90% C.I.)	SRMR	NFI	NNFI	CFI	GFI	AGFI
Single factor — congeneric model	266.54 (2); p < .001	0.35 [0.32–0.39]	0.11	0.81	0.42	0.81	0.89	0.44
Single factor — tau equivalent	394.72 (5); p < .001	0.27 [0.19–0.25]	0.12	0.95	0.70	0.75	0.84	0.97
Single factor — tau equivalent (correlation item 1 and 2; item 3 and 4)	37.81 (3); p > .001	0.10 [0.08–0.14]	0.05	0.97	0.95	0.98	0.98	0.94
Single factor — parallel model	404.01 (8); p > .001	0.22 [0.31–0.44]	0.12	0.73	0.80	0.74	0.84	0.80

#### Discriminant validity

The CoRP had a low correlation with the GHQ (r = 0.14; p ≤ 0.000).

#### Crossover path analysis (test–retest)

To examine the stability of the scale, we performed a crossover path analysis of the CoRP later in time, considering a reduced sample (N = 46). We correlated the time points May 2020 (t0) and June 2020 (t1). Results demonstrated that the initial results predicted those at the follow-up (r = 0.32; p = 0.03).

#### Testing for factor invariance

Testing for the factor invariance of the CoRP scale required several steps. The first step was to perform preliminary confirmatory factor analyses, CFA model, in which single factor (tau equivalent with correlation between item 1 and 2 and item 3 and 4) was posited separately for young (18–35 years; N = 513) and adult samples (36–80 years; N = 508). The model taken into account fitted the data well in each group: young: *X*^2^ = 6.21 (3), *p* < 0.001; CFI = 1.00; NFI = 0.99; RMSEA = 0.046 (0.000 0.097); SRMR = 0.026; AGFI = 0.98; adult: *X*^2^ = 34.50 (3), *p* < 0.001; CFI = 0.95; NFI = 0.94; RMSEA = 0.15 (0.11; 0.20); SRMR = 0.082; GFI = 0.96. Multigroup CFAs were subsequently conducted with the aim to examine Metric Invariance (Thurstone, 1947). The model taken into account fitted the data well: *X*^2^ = 44.70 (6), *p* < 0.001; CFI = 0.97; NFI = 0.97; RMSEA = 0.11 (0.083; 0.14); SRMR = 0.082; GFI = 0.96. Standardized factor loadings of items were all significant. The coefficients have a value of 0.66 for young people and 0.68 for adults.

## Overall discussion and conclusion

Governments and public health authorities urgently need guidance and actionable information on effective public health and psychological interventions that can safeguard the mental health of the general public in the COVID-19 pandemic (Rubin et al., 2020). Risk perception could be a key concept in the prevention of risky behavior and for effectively managing public health risks (Dryhurst et al., 2020). Given the importance of human psychological and behavioral factors in managing pandemics, it is crucial to assess psychological and behavioral responses to the situation to determine how perceived risk is linked to engagement in protective behaviors (Bish & Michie, 2010). Considering the risk to the public that the COVID-19 poses, developing a brief and valid instrument to measure individuals’ risk perception is both timely and important.

This study responded to the very call by identifying relevant content for standardized measures of risk perceptions and examining the structure, reliability, construct validity and invariance of the CoRP, a new measure that assesses people perceptions of the risk to COVID-19. The CoRP, although short, was found to be a robust assessment scale. Findings from the two studies demonstrated that CoRP has a stable unidimensional structure. Also, the scale had strong convergent and discriminant validity. Furthermore, our results contribute to an ongoing debate regarding the utility of short scales for measuring individual differences (Ziegler et al., 2014). The scale can be used to provide valuable information on how individuals perceive COVID-19 risk to institutions and healthcare providers who can design and further improve appropriate prevention programs.

Despite its contributions and the promising results, the present research has some limitations. Firstly, self-reported data could lead to common method variance issues and future studies should take a longitudinal approach to test the models more reliably over time. It would also be important to determine the associations of the scale with non-self-report assessments, and to include actual behavioral measures. The tests for validity were carried out on convenience samples and future research should test whether this result is reproducible in a representative sample. Self-report instruments have the potential for issues of social desirability bias. Although we need to consider this limitation, it is reasonable to think that our data are not highly influenced by this bias because anonymity was guaranteed in data collection (Roccato, 2006).

Based on the strong psychometric properties of the scale, we recommend a wide use of the CoRP in COVID-19 research and health interventions. The scale should also be tested and validated in languages other than Italian for usage across cultures. Governments and public health authorities around the globe should pay a closer attention to the role of risk perception at the individual level in more effectively managing policies and interventions around COVID-19, and for this purpose, we believe the CoRP can prove to be a useful tool.
